# Risk factors for cancer of unknown primary: a literature review

**DOI:** 10.1186/s12885-023-10794-6

**Published:** 2023-04-05

**Authors:** Karlijn E. P. E. Hermans, Fatemeh Kazemzadeh, Caroline Loef, Rob L. H. Jansen, Iris D. Nagtegaal, Piet A. van den Brandt, Leo J. Schouten

**Affiliations:** 1 Department of Epidemiology, GROW School for Oncology and Reproduction, Maastricht University, PO Box 616, 6200 MD Maastricht, the Netherlands; 2 Department of Pathology, Radboud University Medical Centre, Nijmegen, the Netherlands; 3 Department of Research, Comprehensive Cancer Organization the Netherlands, Utrecht, the Netherlands

**Keywords:** Cancer of unknown primary, Risk factors, Review

## Abstract

Cancer of Unknown Primary (CUP) is metastatic cancer with an unidentifiable primary tumour origin during life. It remains difficult to study the occurrence and aetiology of CUP. Hitherto, it is unclear whether risk factors are associated with CUP, yet identifying these factors could reveal whether CUP is a specific entity or a cluster of metastasised cancers from various primary tumour origins. Epidemiological studies on possible CUP risk factors were systematically searched in PubMed and Web of Science on February 1^st^, 2022. Studies, published before 2022, were included if they were observational human-based, provided relative risk estimates, and investigated possible CUP risk factors. A total of 5 case–control and 14 cohort studies were included. There appears to be an increased risk for smoking in relation to CUP. However, limited suggestive evidence was found to link alcohol consumption, diabetes mellitus, and family history of cancer as increased risks for CUP. No conclusive associations could be made for anthropometry, food intake (animal or plant-based), immunity disorders, lifestyle (overall), physical activity, or socioeconomic status and CUP risk. No other CUP risk factors have been studied. This review highlights smoking, alcohol consumption, diabetes mellitus and family history of cancer as CUP risk factors. Yet, there remains insufficient epidemiological evidence to conclude that CUP has its own specific risk factor profile.

**Supplementary Information:**

The online version contains supplementary material available at 10.1186/s12885-023-10794-6.

## Background

Cancer of unknown primary (CUP) is an aggressive unpredictable metastatic cancer with an unidentifiable primary tumour origin during life [1–4]. CUP patients can be categorized into two prognostic subgroups. About 15–20% of the CUP patients reflect a favourable subset, including neuroendocrine carcinomas of unknown primary, peritoneal adenocarcinomatosis of a serous papillary subtype, isolated axillary nodal metastases in females, squamous cell carcinoma involving non-supraclavicular cervical lymph nodes [5]. The disease predominantly occurs in older individuals with a median age of 60 years [6]. The National Institute for Health and Clinical Excellence (NICE) guideline categorised CUP into 1) malignancy of undefined primary origin (MUO), 2) provisional CUP: metastatic epithelial or neuroendocrine malignancy identified based on microscopical verification, and 3) confirmed CUP: metastatic epithelial or neuroendocrine malignancy identified based on final histology, with no primary site detected despite a selected initial screen of investigations, specialist review, and specialised investigations as appropriate [7, 8]. These categories are useful in clinical settings, but population-based research datasets contain a mixture of CUP cases that do not clearly distinguish provisional from confirmed cases. With this mixture [1, 8, 9], variability in disease registrations and diagnostic workup between countries [9–11], it remains hard to compare CUP occurrence globally and identify time trends, as well as assessing its aetiology.

### Cancer risk factors and prevention

The International Agency for Research on Cancer (IARC) Monographs have identified various environmental factors that are carcinogenic hazards to humans, which it continually reviews and updates. These include chemicals, occupational exposures, physical agents, biological agents, and lifestyle factors [12]. Identifying risk factors can guide primary prevention to reduce diseases [13, 14] and for CUP specifically, this is especially important given the bleak prognosis.

### Rationale

To the best of our knowledge, one review examined pointers of disease mechanisms associated with CUP [15], yet, in recent years, the epidemiological evidence regarding those pointers has expanded, which is why we provide here a comprehensive review of current CUP risk factors. We have examined risk factors in association with CUP, considering that a risk factor profile for CUP may imply whether CUP is a specific entity or a cluster of metastasised cancers from various primary tumour origins.

## Materials and methods

The literature search on CUP risk factors was performed in PubMed and Web of Science on February 1^st^, 2022 by using the following keywords (MeSH) and free text terms for the exposure groups: alcohol consumption; anthropometry (body mass index, waist circumference, body constitution and waist-hip ratio); diabetes mellitus (DM); drinks (coffee, caffeine, tea); family history of cancer (FHC) (medical history taking, genetic predisposition to disease); foods (vegetables, fruits, meats, fish products, dairy products, milk, soy milk, eggs, soy foods, soybeans, bread, whole grains, cereal, nuts and seeds); physical activity (exercise, sedentary behaviour); smoking (smoking and tobacco smoke pollution); socioeconomic status (SES) (social conditions, income, poverty, socioeconomic factors, employment, unemployment, work, occupations, education, educational status, health, health insurance, health education, health promotion, health behaviour); racial groups and ethnicity; radiation exposure and environmental pollutants (carcinogens); hormonal factors (estrogens, progesterone, testosterone and oral hormonal contraceptives); and reproductive factors (maternal age, menarche, menopause, post-menopausal hormone replacement therapy, parity), in relation to the outcome: neoplasms of unknown primary, also referred to as cancer of unknown primary (see Additional file 1).

Studies were included if they were observational (e.g., cohort and case–control) human-based, provided risk estimates with *p*-values or 95% confidence intervals, and/or if they had data on at least one of the abovementioned exposure groups. No language restrictions were used. The reference lists of the included articles were checked for potentially relevant studies. Data were extracted for general characteristics and exposure estimates. Due to variability between the studies concerning the study design, different exposures (including differences in exposure measurement), and differences in confounder adjustment, it was not possible to conduct a pooled meta-analysis. Therefore, the existing epidemiological evidence was compared and described as a comprehensive discussion on CUP risk factors. All studies were evaluated against the World Cancer Research Fund’s (WCRF) criteria as epidemiological evidence for cancer prevention, which ranges from convincing to limited-no conclusion. Its criteria are derived from the Bradford Hill criteria which consider the strength of association, temporality, consistency, biological plausibility, dose–response relationship, and experimental evidence [16].

One researcher (K.H.) screened abstracts and eligible full texts, and uncertainties were discussed with a second researcher (L.S.). The reference lists of included articles were checked for additional studies.

## Results

**Fig. 1:**
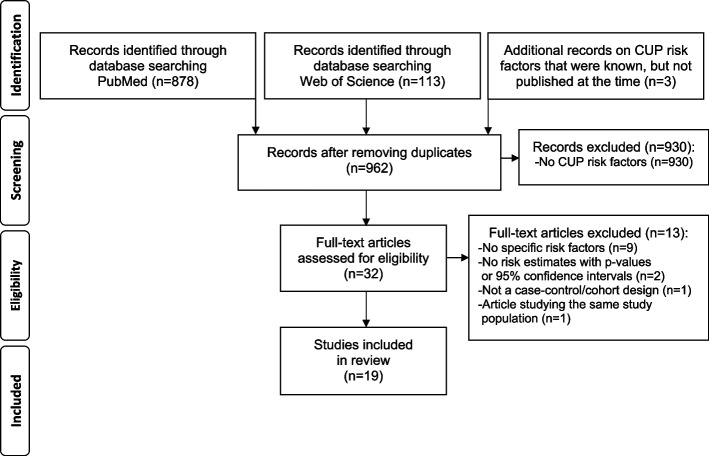
Flowchart of included and excluded studies

The PubMed and Web of Science search yielded 878 and 113 articles, respectively. Three records were additionally identified through other sources, these studies were conducted by our own research group on diabetes mellitus, adherence to lifestyle recommendations for cancer prevention, and vegetable and fruit consumption in relation to CUP risk [17–19]. A total of 930 records were excluded as they did not investigate risk factors associated with CUP. After all exclusions, a total of 19 articles of which were deemed eligible for inclusion (Fig. 1). Overall, eight research teams had examined CUP risk factors in European, American, and Australian populations, representing 5 case–control and 14 cohort studies (Tables 1 and 2). Record linkage methods for exposure and follow-up measurements were applied through country-specific cancer, pathology, and healthcare registers. The search revealed studies on alcohol consumption, anthropometry, DM, FHC, food intake (animal and plant-based), immunity disorders, lifestyle (overall), physical activity, smoking, and SES in relation to CUP risk (Supplementary Tables A, B, C, D, E, F, G, H, I, J and K). No studies had examined the association between drinks, racial groups and ethnicity, radiation exposure and environmental pollutants, hormonal factors, or reproductive factors, and CUP risk.

**Table 1:** General characteristics of case–control studies on risk factors and cancer of unknown primary

Reference	Country	Record linkage	CUP-definition	Assessed exposures
Crawford et al., 2017 [20]	The United Kingdom	Northern and Yorkshire Cancer Registry Information and Service	ICD 10: C80 malignant neoplasm without specification of site	Deprivation
Hemminki et al., 2011,2012 [21, 22]	Sweden	Swedish Family-Cancer Database, MigMed2 dataset at the Center for Primary Health Care Research, Lund University, Malmö	ICD: 7, not further specified	Family history of cancer
Hemminki et al., 2014 [23]	Sweden	Swedish prospective biobanks (Umea Medical Biobank and the Malmo Diet and Cancer Study and the Prevention Study)	ICD: 7, 9, 10, not further specified	BMI, smoking
Hemminki et al., 2016 [24]	Sweden	Swedish healthcare registers: national Hospital Discharge Register (diagnoses 1997–2010), national Outpatient Registry (2001–2010), and the Primary Health Care Registry in Stockholm County (2001–2007). Patients were linked to the Swedish Cancer Registry	CUP diagnoses obtained from Swedish Cancer Registry	Diabetes mellitus

*Abbreviations*: *BMI* Body mass index, *FU* Follow-up, *N.A.* Not applicable

**Table 2:** General characteristics of cohort studies on risk factors and cancer of unknown primary

Reference	Country	Study name	Follow-up	Record linkage	CUP-definition	Assessed exposures
Hemminki et al., 2015 [25]	Sweden	N.A	1964–2012	Swedish Hospital Discharge Register, the KriMed database at Center for Primary Health Care Research, Malmö, Lund University	ICD: 7 code 199, ICD: 9 code 1990–1991	Autoimmune diseases
Kaaks et al., 2014 [10]	Denmark, France, Germany, Greece, Italy, the Netherlands, Norway, Spain, Sweden, and the United Kingdom	The European Prospective Investigation into Cancer and Nutrition Cohort	1992–2000	Linkages with cancer and pathology registries. Health insurance records (France), direct contacts with participants and their next of kin (Germany and Greece); disease occurrence in the latter countries were systematically verified against clinical and pathological records	ICD-O-2: C809: unknown primary site	Alcohol consumption, anthropometry (BMI and waist circumference), levels of education, smoking
Samadder et al., 2016 [26]	Unites States of America	Utah Population Database from the Genealogical Society of Utah	1980–2010	Electronic records for patients with CUP derived from the Utah Cancer Registry	ICD-O-3: C80.9: unknown primary site Histology subtypes: adenocarcinoma (M8140- 8389), squamous cell carcinoma (M8050-8089), carcinoma not otherwise specified (M8010-8049), and neuroendocrine (M8240, 8241, 8243–8246, 8249)	Family history of cancer
Urban et al., 2016 [27]	Unites States of America	Surveillance, Epidemiology and End Results (SEER)-files	SEER 9: 1973–1991 SEER 13: 1992–1999 SEER 17: 2000–2008	SEER-program of the National Cancer Institute in the United States	ICD-O-3: C80.9: unknown primary site Histology subtypes: adenocarcinoma (8140–8389); squamous cell carcinoma (8050–8089); carcinoma not otherwise specified (NOS; 8010–8049) and neuroendocrine (8240, 8241, 8243–8246 and 8249)	SES
Vajdic et al., 2019, 2019 [28, 29]	Australia	The Sax Institute’s 45 and Up Study	1994–2012	NSW Cancer Registry (1994–2012), NSW Admitted Patients Data Collection (2001–2015), NSW Emergency Department Data Collection (2005–2016), and NSW Registry of Births, Deaths, and Marriages (2006–2016) Registry of Births, Deaths, and Marriages (2006–2016)	ICD-O-3: C80 (unknown primary site), C76 (other and ill-defined sites), C26 (other and ill-defined digestive organs) or C39 (other and ill-defined sites within respiratory system and intrathoracic organs) within respiratory system and intrathoracic organs)	Age, alcohol consumption, BMI, meat consumption, physical activity, SES: educational attainment; employment; household income; private health insurance; and residential location, sex, smoking, vegetable and fruit consumption, self-reported health conditions, self-reported family history of cancer, and hospital-recorded health conditions
Pavlidis et al., 2020 [30]	Unites States of America	Surveillance, Epidemiology and End Results (SEER)-files	SEER 9: 1973–1991 SEER 13: 1992–1999 SEER 17: 2000–2008	SEER-program of the National Cancer Institute in the United States	ICD-O-3: C80.9: unknown primary site Histology subtypes: adenocarcinoma (M8140–8239, 8241–8245, 8247, 8248, 8350–8389); squamous cell carcinoma (M8050–8089); carcinoma with neuroendocrine differentiation (M8240, 8246, 8249)	Ethnicity, residential location, SES
Hermans et al., 2020–2022 [17–19, 31–33] & Grewcock et al., 2021 [34]	The Netherlands	The Netherlands Cohort Study	1986–2006	Record linkage with the Netherlands Cancer Registry, Dutch Pathology Registry, and Registration of Municipal Administration	ICD-O-3: C80.9: unknown primary site Histology subtypes: M8000-8570	Alcohol consumption, anthropometry (BMI and clothing size), diabetes mellitus, foods (animal and plant), overall lifestyle, physical activity, smoking & Family history of cancer

### Evaluation of results

Based on the grading criteria in relation to CUP risk, convincing – strong evidence was found for smoking, whereas limited to suggestive evidence was seen for alcohol consumption, DM, and FHC, and limited—no conclusive evidence for anthropometry, food intake (animal or plant-based), immunity disorders, lifestyle (overall), physical activity, or SES (Table 3).

**Table 3:** Exposure evaluation according to the grading criteria as evidence for cancer prevention as reported by the World Cancer Research Fund applied to cancer of unknown primary risk

Exposure	Number of studies & study design	Evaluation of results	Category of evidence^a^
Alcohol consumption	3 Cohort [10, 28, 31]	- Evidence from at least two independent cohort studies - The direction of effect is generally consistent though some unexplained heterogeneity may be present - Evidence for biological plausibility	Limited – suggestive
Anthropometry	1 Case–control [23] 3 Cohort [10, 18, 32]	- Evidence is so limited that no firm conclusion can be made	Limited – no conclusion
Diabetes mellitus	1 Case–control [24] 2 Cohort [18, 29]	- Evidence from at least two independent cohort studies - The direction of effect is generally consistent though some unexplained heterogeneity may be present - Evidence for biological plausibility	Limited – suggestive
Family history of cancer	2 Case–control [21, 22] 3 Cohort [26, 29, 34]	- Evidence from at least two independent cohort studies - The direction of effect is generally consistent though some unexplained heterogeneity may be present - Evidence for biological plausibility	Limited – suggestive
Foods – animal based	2 Cohort [28, 33]	- Evidence is so limited that no firm conclusion can be made	Limited – no conclusion
Foods – plant based	2 Cohort [17, 28]	- Evidence is so limited that no firm conclusion can be made	Limited – no conclusion
Immunity – autoimmune diseases	1 Case–control [25]	- Evidence is so limited that no firm conclusion can be made	Limited – no conclusion
Lifestyle – overall	1 Cohort [19]	- Evidence is so limited that no firm conclusion can be made	Limited – no conclusion
Physical activity	2 Cohort [28, 32]	Evidence is so limited that no firm conclusion can be made	Limited – no conclusion
Smoking	1 Case–control [23] 3 Cohort [10, 28, 31]	- Evidence from more than one study type - Evidence from at least two independent cohort studies - No substantial unexplained heterogeneity within or between study types or in different populations relating to the presence or absence of an association, or direction of effect - Good-quality studies to exclude with confidence the possibility that observed association results from random or systematic error, including confounding, measurement error and selection bias - Presence of a plausible biological gradient (dose–response) in the association. Such a gradient need not be linear or even in the same direction across the different levels of exposure, so long as this can be explained plausibly	Convincing – strong evidence
Socioeconomic status	1 Case–control [20] 3 Cohort [27, 28, 30]	- Evidence is so limited that no firm conclusion can be made	Limited – no conclusion

^a^Category of evidence can be distinguished into the following subgroups as issued by the World Cancer Research Fund: ‘convincing’, ‘probable’, ‘limited-suggestive’, ‘limited-no conclusion’, and ‘substantial effect on risk unlikely’

### Smoking

**Fig. 2:**
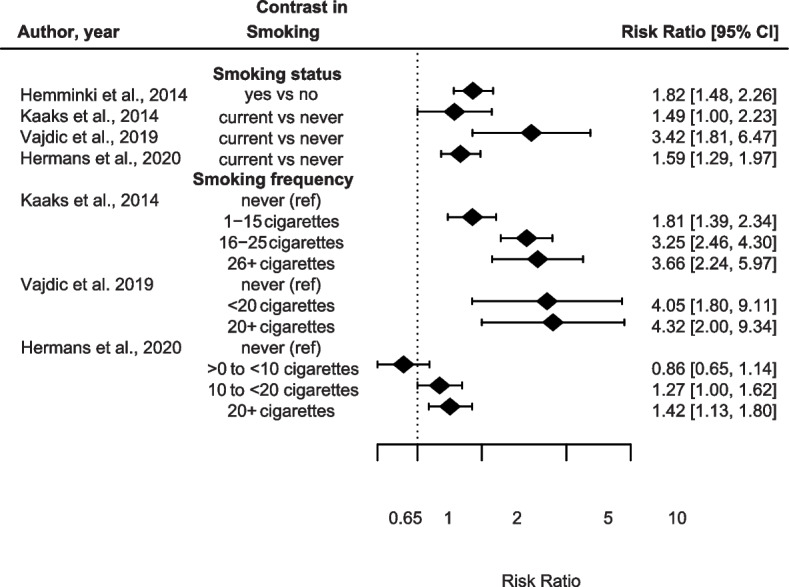
Contrast smoking in relation to CUP risk

Four studies explored the association between smoking and CUP risk (Fig. 2 and Supplementary Table A). All studies reported statistically significantly increased associations for smoking status in relation to CUP [10, 23, 28, 31]. Kaaks et al. & Vajdic et al. also observed an even higher CUP risk among participants who smoked the highest number of cigarettes per day (26 + and 20 + , respectively) compared to never smokers. Similarly, Hermans et al. observed a statistically significant association for smoking frequency which became higher with an increasing number of cigarettes smoked compared to never smokers. They also found smoking duration ≥ 40 years (multivariable adjusted HR: 1.45, 95% CI: 1.09–1.94, *P*trend = 0.02), and smoking cessation (current smokers) associated with increased CUP risk (multivariable adjusted HR: 1.67, 95% CI: 1.37–2.03, *P*trend < 0.001) compared to never smokers [31]. Although the strength of the associations varies between these studies, they all point to a positive association between smoking and CUP risk, particularly in the highest exposure categories.

### Alcohol consumption

**Fig. 3:**
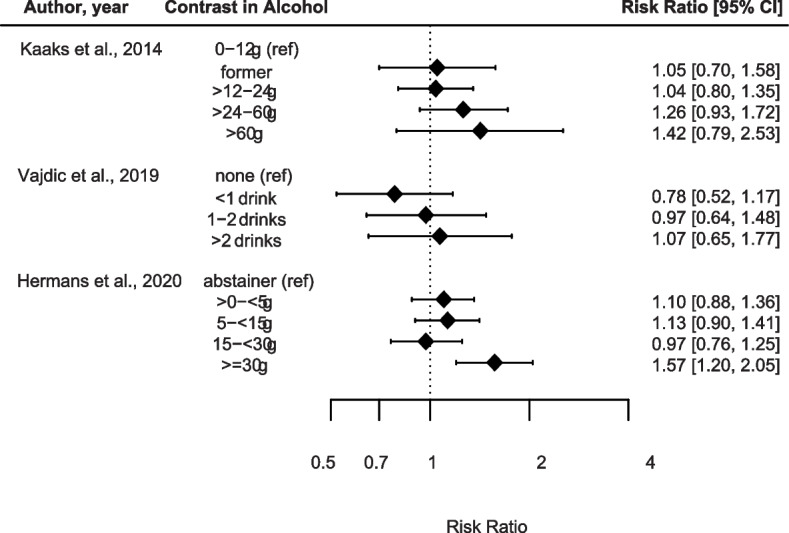
Contrast alcohol consumption in relation to CUP risk

Alcohol consumption related to CUP risk was investigated in three studies (Fig. 3 and Supplementary Table B). Kaaks et al. & Hermans et al. reported increased risks for participants in the highest exposure categories of alcohol consumption > 60 g and ≥ 30 g in relation to CUP compared to 0-12 g and abstainers, respectively [10, 31], whereas Vajdic et al. observed no associations between alcohol consumption and CUP risk compared to non-consumers [28]. Despite the different consumption categories and confounder adjustments there is a suggestive relationship between alcohol consumption and CUP risk.

### Diabetes mellitus

**Fig. 4:**
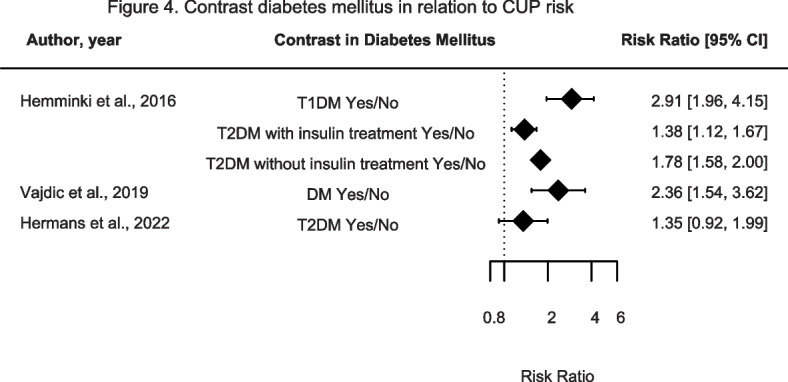
Contrast diabetes mellitus in relation to CUP risk

The association between diabetes mellitus and CUP risk was investigated in three studies (Fig. 4 and Supplementary Table C). Hemminki et al., found that participants with Type 1 (T1DM) and Type 2 DM (T2DM) (with or without insulin treatment) had a statistically significantly increased CUP risk compared to participants without DM [24]. Similarly, Vajdic et al. also found a statistically significant relationship between DM and increased CUP risk compared to participants without DM [29]. Lastly, Hermans et al. observed a non-significant association between T2DM and increased CUP risk compared to participants with no DM [18]. Overall, there appears to be a suggestive association between DM and increased CUP risk. Though its strength might be affected due to inability of confounder adjustment.

### Family history of cancer

**Fig. 5:**
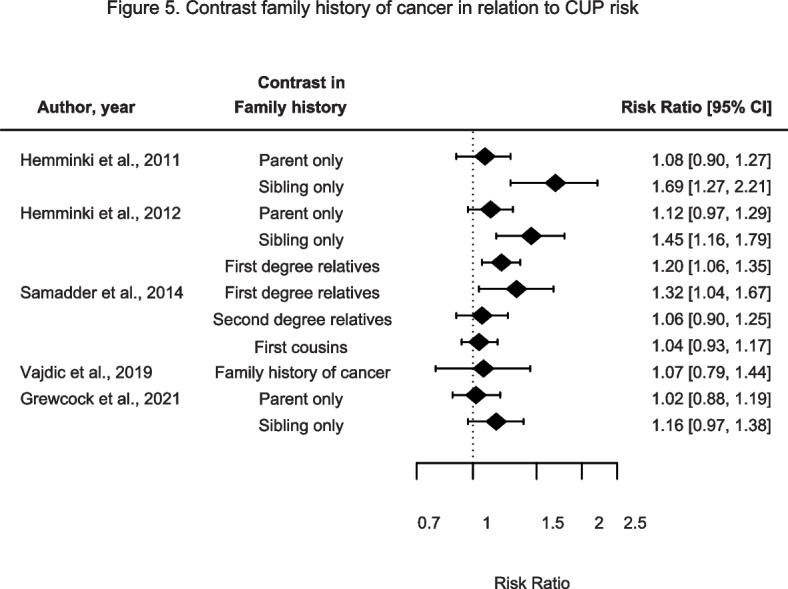
Contrast family history of cancer in relation to CUP risk

Five studies reported on the association between family history of cancer and CUP risk (Fig. 5 and Supplementary Table D). Hemminki et al. found statistically significantly increased CUP risks in siblings alone, while no associations were found between FHC and CUP risk in parents alone [21, 22]. In a follow-up study, Hemminki et al. reported a statistically significantly increased CUP risk in first degree relatives [21]. Similarly, Samadder et al. reported a statistically significant association between family history of cancer and CUP risk in first-degree relatives, but, no associations in second-degree relatives or first cousins [26]. Vajdic et al. reported no association between FHC and CUP risk [29]. Grewcock et al. observed a non-significant increased CUP risk for FHC in siblings only. No associations were found between FHC in parents only in relation to CUP risk [34]. Therefore, there seems to be a suggestive association between FHC and CUP risk. Both Hemminki et al. and Grewcock et al. suggest an association between FHC in siblings only concerning CUP risk, but confounder adjustment was not conducted in the study by Hemminki et al. It is possible that the findings observed for siblings result from a shared environment, which is less likely between the parents and the index-case.

### Anthropometry

Four studies investigated the association between anthropometry and CUP risk (Supplementary Table E). Hemminki et al. compared CUP patients with a BMI ≥ 20 to CUP patients with a BMI < 20 (case–control), and found a decreased CUP risk, albeit not statistically significant [23]. Kaaks et al. found no associations between BMI and CUP risk, but when comparing the highest quartile to the lowest, they did observe that participants with an increasing waist circumference were at an increased CUP risk (multivariable adjusted HR: 1.29, 95% CI: 1.02–1.65, *P*trend = 0.01), which suggests a potential link with abdominal fat [10]. Vajdic et al. noted that obese participants had a non-significant increased CUP risk compared to normal weight participants (age-sex adjusted OR: 1.37, 95% CI: 0.87–2.13) [28]. Hermans et al. explored the association by investigating height (sex-stratified), BMI at baseline, BMI at age 20 years, change in BMI since age 20 years, and clothing size as a proxy for waist circumference (trouser size for men, skirt size for women), but even after multivariable adjustments found no associations between these variables in relation to CUP risk [32].

### Foods (animal-based)

Vajdic et al. and Hermans et al. investigated consuming animal foods and CUP risk (Supplementary Table F). Neither study found any association in respect to red meat consumption. However, Vajdic et al. found an inverse association between processed meat consumption and CUP risk (age-sex adjusted OR: 1.28, 95% CI: 0.82–1.99) compared to consumers < 3 meat per week [28], while Hermans et al. found a statistically significantly increased CUP risk for participants with the highest consumption (Q4) of processed meats compared to the lowest consumption (Q1) categories (multivariable adjusted HR: 1.40, 95% CI: 1.12–1.75, *P*trend = 0.006) [33].

### Foods (plant-based)

Two studies investigated plant foods consumption in relation to CUP risk. Vajdic et al. reported that participants with an intake of ≥ 5 vegetables per day, or an intake of ≥ 2 fruits per day, had a non-significant decreased CUP risk (age-sex adjusted OR: 0.79, 95% CI: 0.57–1.10 & OR: 0.73, 95% CI: 0.53–1.00, respectively) compared to consuming < 5 vegetables per day, and < 2 fruits per day [28]. Hermans et al. studied vegetable and fruit consumption as a group, and as individual components for vegetables, legumes, and fruits, but found no associations between any (Q4) of the plant food exposures in relation to CUP risk compared to the lowest intake (Q1) categories (multivariable adjusted HR: 0.97, 95% CI: 0.78–1.20, *P*trend = 0.63) [17] (Supplementary Table G).

### Physical activity

Two studies have reported on the relationship between physical activity and CUP risk (Supplementary Table H). Vajdic et al. found that participants who were physically active for > 150 min per week (total and moderate-vigorous physical activity) had a statistically significant decreased CUP risk (age-sex adjusted OR: 0.63, 95% CI: 0.44–0.88) compared to participants who were physically active for < 150 min per week. They also found that physically active participants, > 2 times per week, had an even lower CUP risk (age-sex adjusted OR: 0.48, 95% CI: 0.26–0.89) compared to < 1 times per week [28]. Hermans et al. studied non-occupational physical activity in relation to CUP risk but found no association after multivariable adjustment when comparing participants who were physically active for > 90 min per day to ≤ 30 min per day [32].

### Socioeconomic status

In the study by Crawford et al., the researchers found a statistically significantly increased risk between deprivation and CUP when comparing most deprived to those that are least deprived (multivariable adjusted OR: 2.07, 95% CI: 1.85–2.31) [20]. In a different SES study, Urban et al. found neither educational level nor poverty to be associated with CUP risk [27] (Supplementary Table I). Vajdic et al. explored components of SES in relation to CUP risk and found participants without school certificate/qualification to be more at risk (multivariable adjusted OR: 1.69, 95% CI: 1.08–2.64) than participants with any school certificate/ qualification. Additionally, disabled/sick participants, or unemployed, had increased CUP risks. Those who held private health insurance had a decreased CUP risk. In terms of income, participants with a lower income, or who did not report their income, had increased CUP risks [28]. In contrast, Pavlidis et al. reported that participants with a high SES had an increased association for CUP risk (RR: 1.90, 95% CI: 1.50–2.60) compared to those with a low SES [30]. Vajdic et al. suggest that a poor SES measured by education, employment, and access to health services, is associated with increased CUP risk, although these findings may differ between populations. Its authors did not report on adjustments for smoking behaviour or alcohol consumption, while both exposures are linked to SES and may thus play an influential role in the association with CUP. In contrast, Pavlidis et al. in their adjusted analysis, found that participants with a higher SES had a higher CUP risk, while in their unadjusted analysis they found a protective risk. Unfortunately, they did not clarify which variables they had adjusted for in the analysis, so it is impossible to rule out potential correlation between variables.

### Immunity disorders

One case–control study, by Hemminki et al. investigated whether dysfunctions of the immune system in autoimmune diseases are linked to increased CUP risk (Supplementary Table J). It found an overall increased CUP risk for patients diagnosed with autoimmune diseases (SIR: 1.27, 95% CI: 1.22–1.32) [25]. However, the researchers could not control for smoking, which may have influenced the association. Diabetes Mellitus Type 1 is also considered to be an auto-immune disease and was not included in the study of Hemminki et al. [25]. In another publication, the researchers found that Diabetes Mellitus Type 1 is associated with an increased CUP risk (SIR: 2.91, 95% CI: 1.96–4.15) [24].

### Lifestyle (overall)

Hermans et al. examined whether adhering to lifestyle recommendations, as issued by the WCRF and American Institute for Cancer Research in 2018 for cancer prevention helps in decreasing CUP risk. Lifestyle was defined as including a healthy weight, physical activity, and the consumption of plant and animal foods, and alcohol. The highest adherence to lifestyle recommendations was significantly associated with a decreased CUP risk in the age-sex adjusted analysis compared to lowest adherence. However, after adjusting for smoking as well the association attenuated (multivariable adjusted HR: 0.87, 95% CI: 0.70–1.08) [19] (Supplementary Table K).

## Discussion

Based on epidemiological evidence from 4 case–control and 14 cohort studies reviewed here, there is an association between smoking and CUP risk, but evidence for alcohol consumption, DM, and FHC is limited suggestive. The evidence does not allow conclusive associations to be made for anthropometry, food intake (animal or plant-based), immunity disorders, lifestyle (overall), physical activity, or SES.

### Explanation of findings

Autopsy results from CUP patients indicate that primary tumours tend to originate in the lung(s) (5–35%) or pancreas (15–20%), and less often the liver and bile ducts (10–15%), or colon/rectum (3–8%) [35]. These higher occurrences for the lung and pancreas may be reflective of the associations observed with smoking and alcohol consumption. After all, it is known that smoking is strongly associated with lung cancer through deregulated cells, cytokines, and growth factors, which may elevate epithelial apoptosis resistance and ultimately result in mutations [15, 36]. Higher levels of alcohol consumption may be linked to primary tumours of the mouth, pharynx, larynx, oesophagus, liver, pancreas, breast and colorectum [10, 37], the mechanisms underlying cancer development include DNA, protein, and lipid alterations, or damage by acetaldehyde, as well as the carcinogenic metabolite of ethanol, oxidative stress, and alterations to hormonal regulations [38].

For DM, other mechanisms may play a role as patients with T2DM generally have an impaired immune system [39]. Studies have reported that T2DM is related to various types of cancer [40], which may influence the ability of the immune system to suppress a primary tumour, but that the metastasis escaped immune suppression [1, 24, 41]. Similarly, when studying FHC, the role of genetic or environmental risk factors may also be indicative of a specific cancer type. Participants were found to have an increased CUP risk if they had a FHC including kidney, colorectal, lung, pancreatic, myeloma and non-Hodgkin lymphoma [21, 22, 26]. These cancer types may be reflective of the primary tumour origin in the CUP patients.

It remains unclear as to whether CUP is a specific entity, or whether there are specific mechanisms that explain its pattern of metastasis. One of the mechanisms that could explain the absence of indicating a primary tumour origin is, as briefly indicated above, that the immune system was able to remove the primary tumour after metastasis as the primary tumour is recognized, but unable to distinguish features of the metastasis and therefore discard the metastasis in some CUP cases [15, 42]. Studies on CUP immune profiling have shown similar immune profiles compared to immune therapy responsive malignancies [43–45]. In CUP specifically, chromosomal instability (CIN) is not common, which may favor immune checkpoint inhibitors (ICI). CUP patients present individual gene alterations that are implicated in immune-evasion as well as resistance to ICI [46]. Some differences in immune responses to foreign and self-antigens are present throughout life, while others depend on gene expressions and hormone status. These differences may be influenced by gender, early environmental exposures, race, and, for example, systemic inflammatory autoimmune diseases, including Diabetes Mellitus Type 1 [47–49]. In addition, the genes involved in the immune system are under constant evolutionary pressure due to pathogens, environmental conditions, and the relocation of populations [47, 48, 50]. The findings here indicate associations with smoking, alcohol consumption, DM, and FHC in relation to CUP risk, and these risk factors are all known to negatively affect the immune system’s ability to intercept malignant cell development [1, 21, 22, 24, 41, 51, 52]. Similar findings have been found in a study that evaluated immunity disorders in relation to CUP risk [25]. Due to the immune system’s (in)ability to intercept, and the association found between immunity disorders and CUP occurrence, one could speculate that the immune system and CUP incidence are correlated. Another study investigated the genomic mutation of response and resistance to ICI in CUP patients, and observed that the genomic correlates of response and resistance were not prognostic except for CUP patients with a tumor mutational burden > 10 mutations per mega base that had a trend for better outcomes when treated with ICI. Nonetheless, more research is necessary to validate these results [53].

### Implications

This literature review examined various factors and showed that smoking, alcohol consumption, DM, and FHC appear to be associated with CUP risk. The heterogeneous nature of CUP as well as the lack of a specific aetiology suggest that CUP is not a specific entity. Indeed, it is more likely that CUP is a cluster of metastasised cancers, which would explain the variation in both aetiology and immunology. It should, however, be emphasized that only a few studies investigated risk factors for CUP.

### Future CUP studies

A novel approach to study specific aspects of a disease is computational pathology. This approach enables scientists to use sources of information, including patients’ histology data, to extract patterns of cancer. Studies have used artificial intelligence based on both molecular information as well as routine histology slides to investigate the feasibility of predicting the tumour of origin in CUP patients [54]. To administer precision-based medicine it is essential to utilize accurate histopathological and molecular classification of tumors. Cancer cells have an influence on the overall loss of DNA methylation and the acquisition of specific patterns of hypermethylation of certain promoters, which can reversibly and irreversibly alter gene functioning, and thus contribute to cancer progression [55]. This procedure could potentially reduce the extensive diagnostic work-ups that patients undergo. Therefore, future studies into the epidemiological risk factors of CUP besides studying metastatic patterns of cancers with known primaries to learn about the progression model of cancers and combining them with computational pathology predictions for CUP could accelerate the diagnostic process and identify the tumour of origin so as to help personalize therapies [56–59].

### Validity and methodological considerations of the epidemiological findings

CUP risk factors have rarely been studied, most probably due to the lack of a consistent disease definition and because of a general lack of awareness. This dearth of research already makes comparisons hard, but that task is made even harder because those studies that have been done have tended to apply different study designs, used different definitions of the outcome measure (e.g. inclusion of cases without a histopathological confirmation), used different exposure assessments, and differences in availability of confounder data. The lack of confounder data collection restricts confounder adjustments in the analyses, and consequently, associations may have been under- or overestimated. It should also be acknowledged that the participating studies are conducted in Australia, Europe and the United States of America. It may be that other risk factors contribute to CUP occurrence in other continents, which could be interesting for future studies.

## Conclusions

This review has highlighted the influence of a healthy lifestyle on CUP risk, and shown that while there does appear to be an increased risk for smoking, there is only limited suggestive evidence for alcohol consumption, DM, and FHC. No conclusive associations can be made for anthropometry, food intake (animal or plant-based), immunity disorders, lifestyle (overall), physical activity, or SES and CUP risk. Consequently, there is insufficient epidemiological evidence to conclude that CUP has its own specific risk factor profile.

## Supplementary Information

**Additional file 1.** Search strategy – risk factors of cancer of unknown primary

**Additional file 2: Table A.** Results of epidemiological studies on smoking and cancer of unknown primary risk. **Table B.** Results of epidemiological studies on alcohol consumption and cancer of unknown primary risk. **Table C.** Results of epidemiological studies on diabetes mellitus and cancer of unknown primary risk. **Table D.** Results of epidemiological studies on family history of cancer and cancer of unknown primary risk. **Table E.** Results of epidemiological studies on anthropometry and cancer of unknown primary risk. **Table F.** Results of epidemiological studies on foods (animal-based) and cancer of unknown primary risk. **Table G.** Results of epidemiological studies on foods (plant-based) and cancer of unknown primary risk. **Table H.** Results of epidemiological studies on physical activity and cancer of unknown primary risk. **Table I.** Results of epidemiological studies on socioeconomic status and cancer of unknown primary risk. **Table J.** Results of epidemiological studies on immunity disorders and cancer of unknown primary risk. **Table K.** Results of epidemiological studies on lifestyle (overall) and cancer of unknown primary risk.

## Data Availability

All data generated or analysed during this study are included in this published article and its supplementary information files (Additional file 1 and the Supplementary Tables A, B, C, D, E, F, G, H and K).
