# Supplementary material for: Risk factors for cancer of unknown primary: a literature review
Source: BMC Cancer. 2023 Apr 5;23:314. doi: 10.1186/s12885-023-10794-6 (PMC10077635; doi:10.1186/s12885-023-10794-6)
Supplement: Supplementary file 1 — Additional file 1. Search strategy – risk factors of cancer of unknown primary [file 12885_2023_10794_MOESM1_ESM.docx]

**Search strategy – Risk factors of Cancer of Unknown Primary**

**Research Question**

What are risk factors of Cancer of Unknown Primary?

**Databases**

PubMed

**Concept 1: Cancer of Unknown Primary**

Search terms:

cancer unknown primary, MeSH= **Neoplasms, Unknown Primary**

carcinoma unknown primary, MeSH= no hits

metastasis unknown origin, MeSH= **Neoplasms, Unknown Primary**

primary metastatic cancer, MeSH= **Neoplasm Metastasis**

unknown primary tumour, MeSH= **Neoplasms, Unknown Primary**

abbreviation CUP – 25 hits concerning hygienic products and protein, no cancer research

abbreviation MUO – 4 hits concerning chemical components, no cancer research

abbreviation UPT – no hits, no cancer research

MeSH entry terms:

**Neoplasms, Unknown Primary**

Occult Primary Neoplasms

Neoplasms, Occult Primary

Neoplasm, Occult Primary

Occult Primary Neoplasm

Primary Neoplasm, Occult

Primary Neoplasms, Occult

Unknown Primary Neoplasms

Neoplasm, Unknown Primary

Primary Neoplasm, Unknown

Primary Neoplasms, Unknown

Unknown Primary Neoplasm

Unknown Primary Tumors

Primary Tumor, Unknown

Primary Tumors, Unknown

Tumor, Unknown Primary

Tumors, Unknown Primary

Unknown Primary Tumor

Neoplasm Metastasis, Unknown Primary

Unknown Primary Neoplasm Metastasis

**Neoplasm Metastasis**

Neoplasm Metastases

Metastases, Neoplasm

Metastasis, Neoplasm

Metastase

Metastases

Metastasis

Decision:

The use of search term “Neoplasms, Unknown Primary” appears to be the best option to study Cancers of Unknown Primary, the search term “Neoplasm Metastasis” provides irrelevant studies. KH completed an additional check to see if articles that were tagged with ‘Neoplasm Metastasis’ were also discovered with the tag ‘Neoplasms, Unknown Primary’, and found that the articles were indeed found by the latter tag. Therefore, the term ‘Neoplasm Metastasis’ was excluded from the search strategy.

**Concept 2: Risk factors**

Search terms derived from exposure definitions which were utilised in articles that were previously included for studying risk factors of CUP. Terms for foods and beverages derived from a search strategy example by the World Cancer Research Fund. The combined list of exposure definitions is presented below, and includes a variety of terms.

Search terms – MeSH terms:

etiology, risk factor, causality

life style, [lifestyle, healthy]

ethanol, alcoholic beverages

smoking, tobacco smoke pollution

body mass index, waist circumference, body constitution, waist-hip ratio, anthropometry

exercise, sedentary behaviour

diet, cooking, [air pollution, indoor]

sugar sweetened beverages, carbonated beverages, energy drinks

coffee, caffeine

tea

food

vegetable

fruit

fabaceae, vigna

meat, red meat, [diet, western], fish products, fishes, poultry, chickens

dairy products, milk, soy milk

eggs

soy foods, soybeans

bread, whole grains, cereal

nuts, seeds

diabetes mellitus

medical history taking, genetic predisposition to disease

socioeconomic status, social conditions, income, poverty, socioeconomic factors, employment, unemployment, work, occupations, education, educational status

health, [insurance, health], medically uninsured

[education, health], health promotion, health behaviour

racial groups, ethnicity

[exposure, radiation], environmental pollutants, [carcinogens, environmental]

hormones, estrogens, progesterone, testosterone, [contraceptives, oral, hormonal], reproductive history, maternal age, menarche, menopause, [hormone replacement therapy, post menopausal] parity

Notes

If [“] encaptures the tag; the word combination was used as the MeSH search term.

* fabaceae (legumes)

*vigna (beans)

**Search strategy PubMed**

**Concept 1:**

**(neoplasm, unknown primary[MeSH Terms]) OR (neoplasm unknown primary[Title/Abstract])**

**AND**

**Concept 2:**

(((((((((((((((((((((((((((((((((((((((((((((((((((((((((((((((((((((((((((((((((((((((((((((((((((((((((((((((((((((((((((((((((((((((((((((((((((((((((((((((((((((etiology[MeSH Terms]) OR (risk factor[MeSH Terms]) ) OR (causality[MeSH Terms])) OR (life style[MeSH Terms])) OR (lifestyle, healthy[MeSH Terms])) OR (ethanol[MeSH Terms])) OR (alcoholic beverage[MeSH Terms])) OR (smoking[MeSH Terms])) OR (tobacco smoke pollution[MeSH Terms])) OR (body mass index[MeSH Terms])) OR (waist circumference[MeSH Terms])) OR (body constitution[MeSH Terms])) OR (waist-hip ratio[MeSH Terms])) OR (anthropometry[MeSH Terms])) OR (exercise[MeSH Terms])) OR (sedentary behaviour[MeSH Terms])) OR (diet[MeSH Terms])) OR (cooking[MeSH Terms])) OR (air pollution, indoor[MeSH Terms])) OR (sugar sweetened beverages[MeSH Terms])) OR (carbonated beverages[MeSH Terms])) OR (energy drinks[MeSH Terms])) OR (coffee[MeSH Terms])) OR (caffeine[MeSH Terms])) OR (tea[MeSH Terms])) OR (food[MeSH Terms])) OR (vegetable[MeSH Terms])) OR (fruit[MeSH Terms])) OR (fabaceae[MeSH Terms])) OR (vigna[MeSH Terms])) OR (meat[MeSH Terms])) OR (red meat[MeSH Terms])) OR (diet, western[MeSH Terms])) OR (fish products[MeSH Terms])) OR (fishes[MeSH Terms])) OR (poultry[MeSH Terms])) OR (chickens[MeSH Terms])) OR (dairy products[MeSH Terms])) OR (milk[MeSH Terms])) OR (soy milk[MeSH Terms])) OR (eggs[MeSH Terms])) OR (soy foods[MeSH Terms])) OR (soybeans[MeSH Terms])) OR (bread[MeSH Terms])) OR (whole grains[MeSH Terms])) OR (cereal[MeSH Terms])) OR (nuts[MeSH Terms])) OR (seeds[MeSH Terms])) OR (diabetes mellitus[MeSH Terms])) OR (medical history taking[MeSH Terms])) OR (genetic predisposition to disease[MeSH Terms])) OR (socioeconomic status[MeSH Terms])) OR (social conditions[MeSH Terms])) OR (income[MeSH Terms])) OR (poverty[MeSH Terms])) OR (socioeconomic factors[MeSH Terms])) OR (employment[MeSH Terms])) OR (unemployment[MeSH Terms])) OR (work[MeSH Terms])) OR (occupations[MeSH Terms])) OR (education[MeSH Terms])) OR (educational status[MeSH Terms])) OR (health[MeSH Terms])) OR (insurance, health[MeSH Terms])) OR (medically uninsured[MeSH Terms])) OR (health promotion[MeSH Terms])) OR (health behaviour[MeSH Terms])) OR (racial groups[MeSH Terms])) OR (ethnicity[MeSH Terms])) OR (exposure, radiation[MeSH Terms])) OR (environmental pollutants[MeSH Terms])) OR (carcinogens, environmental[MeSH Terms])) OR (hormones[MeSH Terms])) OR (estrogens[MeSH Terms])) OR (progesterone[MeSH Terms])) OR (testosterone[MeSH Terms])) OR (contraceptives, oral, hormonal[MeSH Terms])) OR (reproductive history[MeSH Terms])) OR (maternal age[MeSH Terms])) OR (menarche[MeSH Terms])) OR (menopause[MeSH Terms])) OR (hormone replacement therapy, post menopausal[MeSH Terms])) OR (parity[MeSH Terms])) OR (etiology[Title/Abstract])) OR (risk factor[Title/Abstract])) OR (causality[Title/Abstract])) OR (life style[Title/Abstract])) OR (lifestyle healthy[Title/Abstract])) OR (ethanol[Title/Abstract])) OR (alcoholic beverage[Title/Abstract])) OR (smoking[Title/Abstract])) OR (tobacco smoke pollution[Title/Abstract])) OR (body mass index[Title/Abstract])) OR (waist circumference[Title/Abstract])) OR (body constitution[Title/Abstract])) OR (waist-hip ratio[Title/Abstract])) OR (anthropometry[Title/Abstract])) OR (exercise[Title/Abstract])) OR (sedentary behaviour[Title/Abstract])) OR (diet[Title/Abstract])) OR (cooking[Title/Abstract])) OR (air pollution indoor[Title/Abstract])) OR (sugar sweetened beverages[Title/Abstract])) OR (carbonated beverages[Title/Abstract])) OR (energy drinks[Title/Abstract])) OR (coffee[Title/Abstract])) OR (caffeine[Title/Abstract])) OR (tea[Title/Abstract])) OR (food[Title/Abstract])) OR (vegetable[Title/Abstract])) OR (fruit[Title/Abstract])) OR (fabaceae[Title/Abstract])) OR (vigna[Title/Abstract])) OR (meat[Title/Abstract])) OR (red meat[Title/Abstract])) OR (diet, western[Title/Abstract])) OR (fish products[Title/Abstract])) OR (fishes[Title/Abstract])) OR (poultry[Title/Abstract])) OR (chickens[Title/Abstract])) OR (dairy products[Title/Abstract])) OR (milk[Title/Abstract])) OR (soy milk[Title/Abstract])) OR (eggs[Title/Abstract])) OR (soy foods[Title/Abstract])) OR (soybeans[Title/Abstract])) OR (bread[Title/Abstract])) OR (whole grains[Title/Abstract])) OR (cereal[Title/Abstract])) OR (nuts[Title/Abstract])) OR (seeds[Title/Abstract])) OR (diabetes mellitus[Title/Abstract])) OR (medical history taking[Title/Abstract])) OR (genetic predisposition to disease[Title/Abstract])) OR (socioeconomic status[Title/Abstract])) OR (social conditions[Title/Abstract])) OR (income[Title/Abstract])) OR (poverty[Title/Abstract])) OR (socioeconomic factors[Title/Abstract])) OR (employment[Title/Abstract])) OR (unemployment[Title/Abstract])) OR (work[Title/Abstract])) OR (occupations[Title/Abstract])) OR (education[Title/Abstract])) OR (educational status[Title/Abstract])) OR (health[Title/Abstract])) OR (insurance health[Title/Abstract])) OR (medically uninsured[Title/Abstract])) OR (health promotion[Title/Abstract])) OR (health behaviour[Title/Abstract])) OR (racial groups[Title/Abstract])) OR (ethnicity[Title/Abstract])) OR (exposure radiation[Title/Abstract])) OR (environmental pollutants[Title/Abstract])) OR (carcinogens environmental[Title/Abstract])) OR (hormones[Title/Abstract])) OR (estrogens[Title/Abstract])) OR (progesterone[Title/Abstract])) OR (testosterone[Title/Abstract])) OR (contraceptives oral hormonal[Title/Abstract])) OR (reproductive history[Title/Abstract])) OR (maternal age[Title/Abstract])) OR (menarche[Title/Abstract])) OR (menopause[Title/Abstract])) OR (hormone replacement therapy post menopausal[Title/Abstract])) OR (parity[Title/Abstract])
