# Supplementary material for: Risk factors for cancer of unknown primary: a literature review
Source: BMC Cancer. 2023 Apr 5;23:314. doi: 10.1186/s12885-023-10794-6 (PMC10077635; doi:10.1186/s12885-023-10794-6)
Supplement: Supplementary file 2 — Additional file 2: Table A. Results of epidemiological studies on smoking and cancer of unknown primary risk. Table B. Results of epidemiological studies on alcohol consumption and cancer of unknown primary risk. Table C. Results of epidemiological studies on diabetes mellitus and cancer of unknown primary risk. Table D. Results of epidemiological studies on family history of cancer and cancer of unknown primary risk. Table E. Results of epidemiological studies on anthropometry and cancer of unknown primary risk. Table F. Results of epidemiological studies on foods (animal-based) and cancer of unknown primary risk. Table G. Results of epidemiological studies on foods (plant-based) and cancer of unknown primary risk. Table H. Results of epidemiological studies on physical activity and cancer of unknown primary risk. Table I. Results of epidemiological studies on socioeconomic status and cancer of unknown primary risk. Table J. Results of epidemiological studies on immunity disorders and cancer of unknown primary risk. Table K. Results of epidemiological studies on lifestyle (overall) and cancer of unknown primary risk. [file 12885_2023_10794_MOESM2_ESM.docx]

**Table A. Results of epidemiological studies on smoking and cancer of unknown primary risk.**

| **Smoking** | | | |
| --- | --- | --- | --- |
| **Reference** | **Exposure and *N**** | **Risk estimates (95% CI)** | **Adjusted for** |
| Hemminki  et al.,  2014 | Cases:  290 participants smoked,  173 participants did not smoke  Controls:  1140 smoked,  1212 participants did not smoke | **Smoking status**  Smoking versus no smoking  1.82 (1.48-2.26), *p*-value: 2.8x10^-8^ | - |
| Kaaks  et al.,  2014 | Cohort: 521,448  Cases: 619 | **Smoking intensity**  Never smoked: referent  Current smokers, 1-15 cigarettes: 1.81 (1.39-2.34)  Current smokers, 16-25 cigarettes: 3.25 (2.46-4.30)  Current smokers, 26+ cigarettes: 3.66 (2.24-5.97)  Former smokers, quit ≤10 y: 1.34 (0.99-1.80)  Former smokers, quit >10 y: 1.08 (0.86-1.36)  Current smokers, pipe or cigar: 1.49 (1.00-2.23) | Levels of education, BMI, waist circumference, and average lifetime alcohol consumption |
| Vajdic  et al.,  2019 | Cohort:266,933  Cases: 327  General cohort population controls: 981 | **Smoking status**  Regular tobacco smoking (age-sex adjustment)  General cohort population controls  Never: referent  Former: 2.03 (1.44-2.86)  Current: 4.19 (2.33-7.55)  Current, <20/day 4.05 (1.80-9.11)  Current, ≥20/day 4.32 (2.00-9.34) | Age and sex |
|  |  | Regular tobacco smoking (multivariable adjustment)  General cohort population controls  Never: referent  Former: 1.95 (1.33-2.86)  Current: 3.42 (1.81-6.47) | Age, educational attainment, smoking history, self-rated health, self-reported anxiety, self-reported diabetes, and history of cancer at baseline |
| Hermans  et al.,  2020 | Cohort: 120,852  Subcohort: 4,288  Cases: 963 | **Cigarette smoking status**  Never smokers: referent  Ex-smokers: 1.19 (0.97-1.47)  Current smokers: 1.59 (1.29-1.97)  *p*-trend: <.001 | Age at baseline, sex, alcohol consumption, cigarette smoking frequency, and cigarette smoking duration |
|  |  | **Cigarette smoking frequency**  Never smokers: referent  >0 to <10: 0.86 (0.65-1.14)  10 to <20: 1.27 (1.00-1.62)  ≥20: 1.42 (1.13-1.80)  *p*-trend: 0.003 | Age at baseline, sex, alcohol consumption, current cigarette smoking status, and cigarette smoking duration |
|  |  | **Cigarette smoking duration**  Never smokers: referent  >0 to <20: 0.95 (0.71-1.27)  20 to <40: 1.07 (0.86-1.33)  ≥40: 1.45 (1.09-1.94)  *p*-trend: 0.02 | Age at baseline, sex, alcohol consumption, current cigarette smoking status, cigarette smoking frequency |
|  |  | **Time since cigarette smoking cessation**  Never smokers: referent  Stopped ≥20 y: 0.91 (0.67-1.23)  Stopped 10 to <20 y: 1.06 (0.81-1.38)  Stopped >0 to <10 y: 1.26 (0.99-1.62)  Current smokers: 1.67 (1.37-2.03)  *p*-trend: <.001 | Age at baseline, sex, alcohol consumption, number of cigarette pack-years |

**Table B. Results of epidemiological studies on alcohol consumption and cancer of unknown primary risk.**

| **Alcohol consumption** | | | |
| --- | --- | --- | --- |
| **Reference** | **Exposure and *N**** | **Risk estimates (95% CI)** | **Adjusted for** |
| Kaaks  et al.,  2014 | Cohort: 521,448  Cases: 510 | **Daily alcohol consumption**  Former: 1.05 (0.70-1.58)  0 to 12: referent  >12 to 24: 1.04 (0.80-1.35)  >24 to 60: 1.26 (0.93-1.72)  >60: 1.42 (0.79-2.53)  *p*-trend: 0.15 | Levels of education, BMI, waist circumference, and smoking intensity |
| Vajdic  et al.,  2019 | Cohort:266,933  Cases: 327  General cohort population controls: 981 | **Daily alcohol consumption**  General cohort population controls  None referent  <1 drink: 0.78 (0.52-1.17)  1-2 drinks: 0.97 (0.64-1.48)  >2 drinks: 1.07 (0.65-1.77) | Age and sex |
| Hermans  et al.,  2020 | Cohort: 120,852  Subcohort: 4,288  Cases: 963 | **Daily alcohol consumption**  Abstainers: referent  >0 to <5: 1.10 (0.88-1.36)  5 to <15: 1.13 (0.90-1.41)  15 to <30: 0.97 (0.76-1.25)  ≥30: 1.57 (1.20-2.05)  *p*-trend: 0.02 | Age at baseline, sex, current cigarette smoking status, cigarette smoking frequency, and cigarette smoking duration |

**Table C. Results of epidemiological studies on diabetes mellitus and cancer of unknown primary risk.**

| **Diabetes Mellitus** | | | |
| --- | --- | --- | --- |
| **Reference** | **Exposure and *N**** | **Risk estimates (95% CI)** | **Adjusted for** |
| Hemminki  et al.,  2016 | T1DM Cases: 32,600 | **T1DM status**  SIR: 2.91 (1.96-4.15) | - |
|  | T2DM Cases:178,000 | **T2DM status with insulin treatment**  SIR: 1.38 (1.12-1.67)  **T2DM status without insulin treatment**  SIR: 1.78 (1.58-2.00) |  |
| Vajdic  et al.,  2019 | Cohort:266,933  Cases: 327  General cohort population controls: 981 | **Diabetes status**  General cohort population controls  Yes/no: 2.36 (1.54-3.62) | Age and sex |
| Hermans  et al.,  2022 | Cohort: 120,852  Subcohort: 4,288  Cases: 963 | **T2DM status**  Yes/no: 1.35 (0.92-1.99) | Age at baseline, sex, alcohol consumption, current cigarette smoking status, cigarette smoking frequency, and cigarette smoking duration |

Notes:

Abbreviations: DM: diabetes mellitus (T1DM: Type 1, T2DM: Type 2)

**Table D. Results of epidemiological studies on family history of cancer and cancer of unknown primary risk.**

| **Family history of cancer** | | | |
| --- | --- | --- | --- |
| **Reference** | **Exposure and *N**** | **Risk estimates (95% CI)** | **Adjusted for** |
| Hemminki  et al.,  2011 | Offspring individuals: 9,558,512  Cases: 35,168 | **Family history of cancer - Parent only**  SIR: 1.08 (0.90-1.27) | - |
|  |  | **Family history of cancer** - **Sibling only**  SIR: 1.69 (1.27-2.21) |  |
| Hemminki  et al.,  2012 | Offspring individuals: 9,171 with CUP; 5,506 (60%) had a first-degree relative with any cancer  Cases: 56,049 | **Family history of cancer - Parent only**  SIR: 1.12 (0.97-1.29) | - |
|  |  | **Family history of cancer - Sibling only**  SIR: 1.45 (1.16-1.79) |  |
|  |  | **Family history of cancer - First degree relatives**  SIR: 1.20 (1.06-1.35) |  |
| Samadder et al.,  2015 | Cases: 4,160  Controls, non-CUP: 52,036  Controls, cancer-free: 51,053 | **Family history of cancer - First-degree relatives**  cancer-free controls: 1.32 (1.04-1.67) | - |
|  |  | **Family history of cancer - Second-degree relatives**  cancer-free controls: 1.06 (0.90-1.25) |  |
|  |  | **Family history of cancer - First cousins**  cancer-free controls: 1.04 (0.93-1.17) |  |
| Vajdic  et al.,  2019 | Cohort:266,933  Cases: 327  General cohort population controls: 981 | **Family history of cancer**  General cohort population controls  Yes/no: 1.07 (0.79-1.44) | Age and sex |
| Grewcock et al.,  2021 | Cohort: 120,852  Subcohort: 4,288  Cases: 963 | **Family history of cancer – Parent only**  HR: 1.02 (0.88-1.19) | Age at baseline, sex, alcohol consumption, current cigarette smoking status, cigarette smoking frequency, and cigarette smoking duration |
|  |  | **Family history of cancer – Sibling only**  HR: 1.16 (0.97-1.38) |  |

**Table E. Results of epidemiological studies on anthropometry and cancer of unknown primary risk.**

| **Anthropometry** | | | |
| --- | --- | --- | --- |
| **Reference** | **Exposure and *N**** | **Risk estimates (95% CI)** | **Adjusted for** |
| Hemminki  et al.,  2014 | Cases: 418 BMI ≥20, 29 BMI <20  Controls: 2203 BMI ≥20, 113 BMI <20 | **BMI**  BMI ≥20 versus BMI <20 (All CUP)  0.77 (0.50-1.18), *p*-value: 0.23 | - |
| Kaaks  et al.,  2014 | Cohort: 521,448  Cases BMI: 634  Cases waist circumference: 600 | **BMI**  Quartile 1: referent  Quartile 2: 0.92 (0.73-1.16)  Quartile 3: 0.98 (0.78-1.23)  Quartile 4: 1.06 (0.84-1.33)  *p*-trend: 0.29 | Smoking intensity, average lifetime alcohol consumption, and levels of education |
|  |  | **Waist circumference**  Quartile 1: referent  Quartile 2: 0.91 (0.71-1.16)  Quartile 3: 1.02 (0.80-1.30)  Quartile 4: 1.29 (1.02-1.65)  *p*-trend: 0.01 |  |
| Vajdic  et al.,  2019 | Cohort:266,933  Cases: 327  General cohort population controls: 981 | **BMI**  General cohort population controls  Underweight: 2.13 (0.52-8.61)  Normal weight: referent  Overweight: 0.89 (0.61-1.31)  Obese: 1.37 (0.87-2.13) | Age and sex |
| Hermans  et al.,  2020 | Cohort: 120,852  Subcohort: 4,099  Cases: 926 | **Height (men only)**  <170: referent  170-<175: 0.90 (0.65-1.24)  175-<180: 0.85 (0.61-1.19)  180-<185: 0.88 (0.61-1.27)  ≥185: 0.91 (0.59-1.41)  *p*-trend: 0.67  **Height (women only)**  <160: referent  160-<165: 0.74 (0.51-1.06)  165-<170: 0.77 (0.54-1.09)  170-<175: 1.03 (0.69-1.51)  ≥175: 0.99 (0.59-1.67)  *p*-trend: 0.62 | Age at baseline, sex, alcohol consumption, current cigarette smoking status, cigarette smoking frequency, and cigarette smoking duration, and weight |
|  |  | **BMI at baseline**  <20: 0.91 (0.58-1.42)  20-<25: referent  25-<30: 0.90 (0.77-1.06)  ≥30: 1.11 (0.81-1.52)  *p*-trend: 0.77 | Age at baseline, sex, alcohol consumption, current cigarette smoking status, cigarette smoking frequency, and cigarette smoking duration, and non-occupational physical activity |
|  |  | **BMI at age 20 years**  <20: 0.89 (0.71-1.13)  20-<21.5: referent  21.5-<23: 0.93 (0.74-1.17)  23-<25: 0.96 (0.75-1.23)  ≥25: 1.06 (0.85-1.32)  *p*-trend: 0.25 | Age at baseline, sex, alcohol consumption, current cigarette smoking status, cigarette smoking frequency, and cigarette smoking duration, and non-occupational physical activity |
|  |  | **Change in BMI since age 20 years**  <0: 1.24 (0.97-1.61)  0-<4: referent  4-<8: 0.82 (0.68-0.99)  ≥8: 1.04 (0.86-1.26) | Age at baseline, sex, alcohol consumption, current cigarette smoking status, cigarette smoking frequency, and cigarette smoking duration, non-occupational physical activity, and BMI at age 20 years |
|  |  | **Clothing size (proxy for waist circumference)**  Clothing size men (trouser size)  <50: 0.94 (0.68-1.30)  50-51: referent  52-53: 0.82 (0.62-1.08)  54-55: 0.76 (0.54-1.05)  >56: 0.93 (0.68-1.28)  *p*-trend: 0.46  Clothing size women (skirt size)  <40: 0.91 (0.63-1.30)  42: referent  44: 0.99 (0.72-1.36)  46-48: 0.87 (0.62-1.22)  >50: 1.51 (0.91-2.52)  *p*-trend: 0.55 | Age at baseline, sex, alcohol consumption, current cigarette smoking status, cigarette smoking frequency, and cigarette smoking duration, and non-occupational physical activity |

Notes:

Abbreviations: BMI: body mass index

**Table F. Results of epidemiological studies on foods (*animal-based*) and cancer of unknown primary risk.**

| **Animal foods** | | | |
| --- | --- | --- | --- |
| **Reference** | **Exposure and *N**** | **Risk estimates (95% CI)** | **Adjusted for** |
| Vajdic  et al.,  2019 | Cohort:266,933  Cases: 327  General cohort population controls: 981 | **Red meat consumption**  General cohort population controls  ≥3 meat/week: 0.95 (0.69-1.32) | Age and sex |
|  |  | **Processed meat consumption**  General cohort population controls  ≥3 meat/week: 1.28 (0.82-1.99) |  |
| Hermans  et al.,  2021 | Cohort: 120,852  Subcohort: 4,111  Cases: 899 | **Red meat (overall)**  Q1: referent  Q2: 1.11 (0.89-1.37)  Q3: 1.21 (0.98-1.49)  Q4: 1.04 (0.83-1.30)  *p*-trend: 0.31 | Age at baseline, sex, current cigarette smoking status, cigarette smoking frequency, cigarette smoking duration, and total energy intake |
|  |  | **Poultry**  C1: referent  C2: 1.13 (0.91-1.41)  C3: 1.10 (0.88-1.37)  C4: 0.97 (0.79-1.21)  *p*-trend: 0.28 |  |
|  |  | **Processed meat**  Q1: referent  Q2: 1.07 (0.86-1.33)  Q3: 1.14 (0.91-1.42)  Q4: 1.40 (1.12-1.75)  *p*-trend: 0.006 |  |
|  |  | **Fish**  Q1: referent  Q2: 1.30 (1.04-1.61)  Q3: 1.23 (1.00-1.51)  Q4: 1.25 (0.99-1.57)  *p*-trend: 0.29 |  |

**Table G. Results of epidemiological studies on foods (*plant-based*) and cancer of unknown primary risk.**

| **Plant foods** | | | |
| --- | --- | --- | --- |
| **Reference** | **Exposure and *N**** | **Risk estimates (95% CI)** | **Adjusted for** |
| Vajdic  et al.,  2019 | Cohort:266,933  Cases: 327  General cohort population controls: 981 | **Vegetable consumption**  General cohort population controls  ≥5 vegetables/day: 0.79 (0.57-1.10) | Age and sex |
|  |  | **Fruit consumption**  General cohort population controls  ≥2 fruits/day: 0.73 (0.53-1.00) |  |
| Hermans  et al.,  2021 | Cohort: 120,852  Subcohort: 4,005  Cases: 867 | **Total vegetables and fruits (combined)**  Q1: referent  Q2: 1.02 (0.83-1.27)  Q3: 0.96 (0.78-1.19)  Q4: 0.97 (0.78-1.20)  *p*-trend: 0.63 | Age at baseline, sex, current cigarette smoking status, cigarette smoking frequency, and cigarette smoking duration |
|  |  | **Total vegetables**  Q1: referent  Q2: 0.94 (0.76-1.17)  Q3: 1.04 (0.84.-1.28)  Q4: 0.87 (0.69-1.09)  *p*-trend: 0.38 | Age at baseline, sex, current cigarette smoking status, cigarette smoking frequency, cigarette smoking duration, and total fruit consumption |
|  |  | **Legumes**  Q1: referent  Q2: 1.11 (0.90-1.38)  Q3: 1.08 (0.87.-1.35)  Q4: 1.21 (0.97-1.52)  *p*-trend: 0.14 | Age at baseline, sex, current cigarette smoking status, cigarette smoking frequency, cigarette smoking duration, and total vegetable and fruit consumption |
|  |  | **Total fruits**  Q1: referent  Q2: 0.94 (0.76-1.16)  Q3: 0.92 (0.74.-1.15)  Q4: 0.94 (0.75-1.17)  *p*-trend: 0.56 | Age at baseline, sex, current cigarette smoking status, cigarette smoking frequency, cigarette smoking duration, and total vegetable consumption |

**Table H. Results of epidemiological studies on physical activity and cancer of unknown primary risk.**

| **Physical activity** | | | |
| --- | --- | --- | --- |
| **Reference** | **Exposure and *N**** | **Risk estimates (95% CI)** | **Adjusted for** |
| Vajdic  et al.,  2019 | Cohort:266,933  Cases: 327  General cohort population controls: 981 | **Total and moderate vigorous physical activity**  General cohort population controls  >150 min/week: 0.63 (0.44-0.88) | Age and sex |
|  |  | **Total physical activity**  General cohort population controls  <1 times/week: referent  1-2 times/week: 0.95 (0.38-2.33)  >2 times/week: 0.48 (0.26-0.89) |  |
| Hermans  et al.,  2020 | Cohort: 120,852  Subcohort: 4,099  Cases: 926 | **Non-occupational physical activity in minutes per day**  ≤30: referent  >30-60: 0.91 (0.74-1.12)  >60-90: 0.85 (0.67-1.08)  >90: 0.97 (0.78-1.20)  *p*-trend: 0.84 | Age at baseline, sex, alcohol consumption, current cigarette smoking status, cigarette smoking frequency, cigarette smoking duration, and BMI at baseline |

**Table I. Results of epidemiological studies on socioeconomic status and cancer of unknown primary risk.**

| **Socioeconomic status** | | | |
| --- | --- | --- | --- |
| **Reference** | **Exposure and *N**** | **Risk estimates (95% CI)** | **Adjusted for** |
| Crawford  et al.,  2017 | Cases: 7428  Controls: 8849 (C77-C79) and 10,804 (C20) | **Deprivation (Index of Multiple Deprivation)**  Quartile 1 (least deprived): referent  Quartile 2: 1.53 (1.37-1.17)  Quartile 3: 1.74 (1.56-1.94)  Quartile 4: 2.07 (1.85-2.31) | Gender, age group, deprivation quartile and travel time to general practitioner quartile |
| Urban  et al.,  2013 | Cohort: >2,800,000  Cases: 51,294 | **Educational level**  Lowest education: referent  Second quartile: 0.96 (0.94-0.99)  Third quartile: 0.97 (0.95-1.00)  Highest education: 0.95 (0.93-0.98) | - |
|  |  | **Poverty**  Affluent: referent  Middle: 1.02 (1.00-1.04)  Poor: 1.03 (0.98-1.07) |  |
| Vajdic  et al.,  2019 | Cohort:266,933  Cases: 327  General cohort population controls: 981 | **Educational attainment (6-categories)**  General cohort population controls  No school certificate or qualification: referent  School of intermediate certificate: 0.47 (0.29-0.75)  Higher school or leaving certificate: 0.49 (0.26-0.94)  Trade/apprenticeship: 0.75 (0.44-1.29)  Certificate/diploma: 0.50 (0.30-0.85)  University degree or higher: 0.51 (0.30-0.86) | Age and sex |
|  |  | **Employment**  General cohort population controls  Full-time, part-time, or self-employed: 0.85 (0.56-1.28)  Fully or part retired: 0.85 (0.58-1.27)  Disabled/sick: 3.33 (1.67-6.65)  Doing unpaid work: 0.63 (0.30-1.34)  Studying: 1.50 (0.35-6.40)  Looking after home/family: 0.92 (0.52-1.61)  Unemployed: 2.71 (1.01-7.31) |  |
|  |  | **Hold private health insurance**  General cohort population controls  Yes: 0.62 (0.45-0.85) |  |
|  |  | **Residential location**  Major city: referent  Inner regional: 1.01 (0.73-1.41)  Outer regional or rural: 1.46 (0.90-2.35)  Not reported: 0.59 (0.17-1.98) |  |
|  |  | **Yearly household income**  General cohort population controls  $70,000 or more: referent  $50,000-$69,999: 1.38 (0.67-2.85)  $20,000-$49,999: 1.30 (0.73-2.30)  <$20,000: 1.91 (1.08-3.37)  Not reported: 1.79 (1.00-3.21) |  |
|  |  | **Educational attainment (2-categories)**  General cohort population controls  Any school certificate or qualification: referent  No school certificate or qualification: 1.69 (1.08-2.64) | Age, educational attainment, smoking history, self-rated health, self-reported anxiety, self-reported diabetes, and history of cancer at baseline |
| Pavlidis  et al.,  2020 | Cases: 907 adolescents and young adults | **Ethnicity**  Black: 1.17 (1.00-1.40)  Other: 1.01 (0.80-1.20)  White: Reference | **Unclear which variables are adjusted for in the analysis* |
|  |  | **Urban**  Nonmetropolitan: 0.16 (0.10-0.20)  Metropolitan: Reference |  |
|  |  | **Socioeconomic status**  High: 1.90 (1.50-2.60)  Low: Reference |  |

Notes:

Abbreviations: SES: socioeconomic status

**Table J. Results of epidemiological studies on immunity disorders and cancer of unknown primary risk.**

| **Immunity disorders** | | | |
| --- | --- | --- | --- |
| **Reference** | **Exposure and *N**** | **Risk estimates (95% CI)** | **Adjusted for** |
| Hemminki  et al.,  2015 | Cohort: 789,681  Cases: 2,658 | **Autoimmune diseases**  All: 1.27 (1.22-1.32)  Addison’s disease: 1.77 (1.07-2.78)  Celiac disease: 1.55 (1.14-2.05)  Crohn’s disease: 1.59 (1.37-1.83)  Graves’/hyperthyroidism: 1.28 (1.15-1.42)  Myasthenia gravis: 1.43 (1.23-1.65)  Pemicious anemia: 1.55 (1.29-1.84)  Polymyalgia rheumatica: 1.21 (1.05-1.38)  Polymyositis//dermatomyositis: 3.51 (2.44-4.89)  Primary biliary cirrhosis: 1.81 (1.05-2.91)  Psoriasis: 1.15 (1.01-1.30)  Rheumatoid arthritis: 1.14 (1.05-1.25)  Sjögren’s syndrome: 1.55 (1.21-1.96)  Systemic lupus erythematosus: 1.57 (1.15-2.09)  Systemic sclerosis: 1.60 (1.16-2.14)  Ulcerative colitis: 1.54 (1.34-1.77) | - |

**Table K. Results of epidemiological studies on lifestyle (overall) and cancer of unknown primary risk.**

| **Lifestyle (overall)** | | | |
| --- | --- | --- | --- |
| **Reference** | **Exposure and *N**** | **Risk estimates (95% CI)** | **Adjusted for** |
| Hermans  et al.,  2022 | Cohort: 120,852  Subcohort: 3,911  Cases: 856 | **Overall adherence to lifestyle recommendations on cancer prevention; including healthy weight, physical activity, plant and animal foods, and alcohol consumption**  Lowest adherence: referent  Medium adherence: 1.08 (0.90-1.31)  Highest adherence: 0.87 (0.70-1.08)  *p*-trend: 0.99 | Age at baseline, sex, current cigarette smoking status, cigarette smoking frequency, cigarette smoking duration, and total energy intake |
